# Antimicrobial Peptoids Show Potent Antifungal Activity against *Candidozyma (Candida) auris In Vivo* and Broad-Spectrum Activity *In Vitro* against Other *Candida* Species

**DOI:** 10.1021/acsinfecdis.6c00525

**Published:** 2026-09-04

**Authors:** Lisa K. Ryan, Isabella M. Griffiths, Uyen Emily Do, Denny Gao, Erika L. Figgins, Kristian Sørensen, Jennifer S. Lin, Natalia Molchanova, Kent Kirshenbaum, Annelise E. Barron, Gill Diamond

**Affiliations:** † 162193 University of Louisville Center for Predictive Medicine, Louisville, Kentucky 40202, United States; ‡ Department of Oral Immunology and Infectious Diseases, University of Louisville School of Dentistry, Louisville, Kentucky 40202, United States; § Department of Medicine, Division of Infectious Diseases and Global Medicine, University of Florida College of Medicine, Gainesville, Florida 32608, United States; ∥ Department of Bioengineering, 6429 Stanford University Schools of Medicine and Engineering, Stanford, California 94305, United States; ⊥ Molecular Foundry, 682790 Lawrence Berkeley National Laboratory, Berkeley, California 94720, United States; # Department of Chemistry, New York University, New York, New York 10003, United States

**Keywords:** antifungal, nonalbicans, synergy, peptoid, candidemia

## Abstract

Invasive candidiasis is a serious fungal infection caused by *Candida* species that can enter the bloodstream and affect major organs, particularly in immunocompromised patients. Development of resistance to standard antifungal therapies, especially with *C*. *auris*, has become a major public health issue. While antimicrobial peptides such as the human cathelicidin, LL-37, could represent a possible therapeutic strategy, they are often inactivated when delivered systemically. One alternative therapeutic strategy is antimicrobial peptoids. We studied *in vitro* activity of three peptoid compounds, oligomers of *N*-substituted glycines, which mimic the activity of LL-37. Our aim was to establish these peptoid compounds as a potential treatment of invasive candidiasis and compare them to LL-37′s antifungal activity. *In vitro*, LL-37 did not exhibit antifungal activity (MIC > 64) against all *C*. *auris* strains and three species of *Candida*. In contrast, three peptoids were tested against ten *C*. *auris* strains and six other *Candida* species and showed good activity. MICs measured as low as 4 μg/mL for TM5 against *C*. *auris* strain 381. Mechanistic studies using TEM and fluorescent confocal microscopy of peptoids against *C*. *auris* clinical isolates showed membrane disruption and consolidation of organelles. Peptoids TM4 and TM5 showed strong synergistic effects with fluconazole and caspofungin, and amphotericin B. Serial daily passage of TM4 and TM5 at sub-MIC concentrations showed no resistance developing for up to 3 weeks. Fungicidal kinetics revealed complete killing of fungal colonies after around 1 h. A systemic candidiasis mouse model treated subcutaneously with TM5 3 h after *C*. *auris* infection resulted in clearing of infection, indicated by colony forming units (cfu) per gram of kidney, at 24 h with the 5 mg/kg dose. This demonstrates that antimicrobial peptoids are active systemically and have potential to be an antifungal treatment for candidemia, particularly as an alternative therapy for resistant strains.

## Graphical abstract

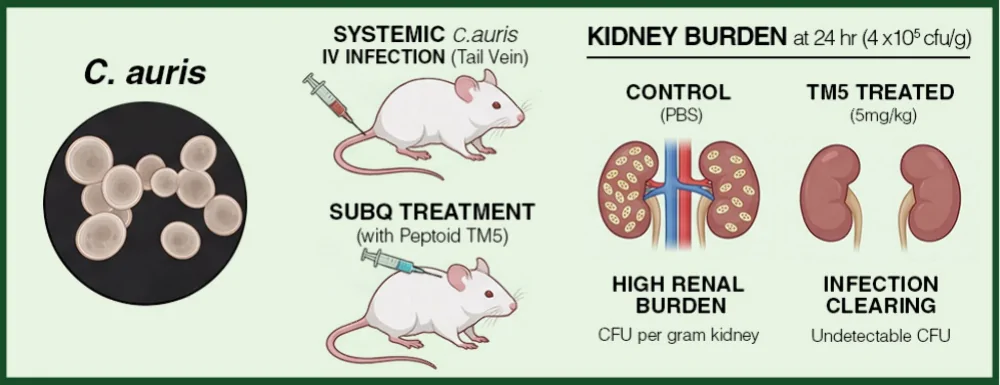

## Introduction

*Candida* species are the most common of systemic and invasive fungal infections. It is estimated that 482,000 die annually from invasive candidiasis worldwide.^1^ *Candida* species are dimorphic, forming as mycelia or yeast cells in the tissues.^2^ A species of great concern in the medical community at present is *Candidozima* (formerly *Candida*) *auris*. The incidence of infection with *C*. *auris* is growing yearly. In 2024, the number of cases in the U.S. was 6,304, primarily nosocomial, and had increased every year from the 744 cases reported to the CDC in 2020.^3^ While endemic to certain locations and not an epidemic, concern is growing due to the resistance profile of *C*. *auris* and the mortality of this systemic fungal infection.^2^ The mortality rate of invasive bloodstream candidiasis due to hospital-acquired *C*. *auris* was reported to be as high as 72%.^2^

*C*. *auris* falls into the XDR (extreme drug resistance) category, meaning patients infected with some strains are resistant to three of the antifungals used to treat the systemic infection.^4^ The echinocandin and fluconazole classes of antifungals are the only two first-line monotherapeutic drugs for invasive candidiasis, followed by amphotericin B. Most *C*. *auris* strains have developed resistance to fluconazole and some to the echinocandins, such as caspofungin. From 3 to 10% of isolates from patients are also resistant to amphotericin B, but this drug also has toxic renal side effects at high doses and is reserved for azole and echinocandin-resistant strains.^5^ The resistance mechanisms of these drugs are reviewed in Hossain et al. 2022.^4^ Complicating the problem is the increase in the number of immunocompromised patients due to new treatments increasing survival from serious diseases and transplantation.^2^ With standard fungistatic antifungal drugs, eradication depends on the immune system functioning well. Thus, it is imperative that new antifungal drugs be developed that bypass toxicity and are slow to develop resistance against *C*. *auris*.

The sensitivity of *Candida* species to antimicrobial peptides (AMPs) has been well documented, especially with the AMP classes of defensins and histatins.^6–10^ Histatin-5, found in human saliva, has broad and potent activity against *C*. *auris*, although *C*. *auris* has a higher tolerance to oxidative stress caused by histatin-5 than does *C*. *albicans*.^11^ Recently, the α-defensin, human neutrophil peptide-1 (HNP-1), and the β-defensin, human β-defensin-3 (hBD-3) were shown to induce both early and late apoptosis, along with significant mitochondrial membrane depolarization resulting in cytochrome C release in *C*. *auris*. In addition, the study showed that histatin-5 caused significant necrosis of *C*. *auris*.^12^ The human cathelicidin, LL-37, has shown activity against *C*. *albicans*, although it is not as potent as some of the other AMPs in fungicidal activity and prevents infection by inhibiting cell adhesion and aggregation.^13^

Host defense peptides are suggested for development of antifungal agents due to their greatly reduced ability to confer resistance,^14^ however, they are subject to degradation by proteases, reducing their efficacy. One line of research inspired by AMPs is the development of antimicrobial peptoids. Antimicrobial peptoids, which are antimicrobial peptide mimic oligomers that possess an *N*-substituted glycine backbone and are efficiently synthesized,^15,16^ and have an advantage over AMPs in that they are resistant to degradation by proteases.^17^ Like AMPs, they possess broad-spectrum antimicrobial efficacy^18^ and are slow to induce resistance in a variety of microorganisms, including *Pseudomonas aeruginosa*,^19^ methicillin-resistant *Staphylococcus aureus* (MRSA),^20^ the intracellular bacterium, *Mycobacterium tuberculosis*,^21^ and the parasite, *Cryptosporidium parvum*.^22^ Several studies have demonstrated disruption of bacterial membranes,^23,24^ biofilms^25^ and viral envelopes.^26–28^ In addition, these peptoids have also been observed to nonspecifically aggregate intracellular contents of *Escherichia coli* ^29^ and *C*. *albicans* ^30^ and inhibit the formation of fungal hyphae in *C*. *albicans*.^30^ In addition, the antimicrobial peptoid TM1 flocculates ribosomes and DNA of *E*. *coli*.^31^

Recent studies have examined the efficacy of antimicrobial peptoids in *Candida* species, including *C*. *auris*.^32–34^ These studies have reported killing of these yeasts *in vitro*. In this study, the sensitivity of several species of *Candida*, including five clades of *C*. *auris*, were tested against several peptoids that were designed and modeled after the structure and activity of LL-37. The mechanism of action of the most efficacious peptoid, TM5 (H-*N*tridec-*N*Lys-*N*spe-*N*spe-*N*Lys-NH_2_ (MW = 835.19),^26^ was then investigated, and resistance studies were conducted with comparison to TM4. In addition, the data show that TM4 and TM5 synergize with the commonly utilized antifungal drugs caspofungin, fluconazole and amphotericin B. Most importantly, we show that TM5 peptoid has fungicidal activity systemically *in vivo* in a mouse model of candidemia induced by *C*. *auris* infection.

## Results

### Minimal Inhibitory Concentrations (MICs) and Minimal Fungicidal Concentrations (MFCs)

An initial study of peptoid activity against *Candida* species was performed. Table 1 shows the killing of 10 strains of *C*. *auris* by three peptoids, TM4, TM5 and TM9 (for structures, see^26^). The 10 *C*. *auris* strains were 381, 382, 383, 384, 385, 386, 387, 388, 389, and 390.

**Table 1:** Minimum Inhibitory Concentration (MIC) and Minimum Fungicidal Concentrations (MFC) for Peptoids TM4, TM5, TM9, and LL-37 against Different Clinical Strains of *Candida auris* in μg/mL

*Strain*	381		382		383		384		385		386		387		388		389		390	
*Peptoid*	MIC	MFC	MIC	MFC	MIC	MFC	MIC	MFC	MIC	MFC	MIC	MFC	MIC	MFC	MIC	MFC	MIC	MFC	MIC	MFC
*TM*4	8	32	8	64	8	32	4	32	8	64	8	64	16	32	8	64	8	32	8	64
*TM*5	4	64	8	>64	8	>64	8	>64	8	64	8	>64	16	>64	8	>64	8	>64	8	>64
*TM*9	8	32	64	>64	8	32	8	16	8	32	8	32	16	64	8	32	8	32	8	64
*LL-*37	>64	>64	>64	>64	>64	>64	>64	>64	>64	>64	>64	>64	>64	>64	>64	>64	>64	>64	>64	>64

The MIC against strain 381 with TM5 was 4 μg/mL (4.8 μM). The MIC using TM4 and TM9 on strain 381 was 8 μg/mL (6.4 μM and 6.2 μM respectively). However, the MFC for TM5 was 64 μg/mL (76.8 μM) and 32 μg/mL for TM4 and TM9 (25.6 μM and 24.8 μM, respectively), showing that *in vitro* killing was not complete with these peptoids. The MIC using strain 382 was 8 μg/mL for peptoids TM4 and TM5. The MIC with TM9 against strain 382 was 64 μg/mL. *C*. *auris* strains 381 and 382 were studied further. In contrast, LL-37 exhibited no activity (MIC > 64; > 14.4 μM) against *C*. *auris* strains 381 and 382.

Table 2 shows the MICs and MFCs of the peptoids on five species of *Candida*. *C*. *krusei* was the most sensitive to the peptoids, with TM5 giving a 25 ng/mL MIC (0.024 μM). As with *C*. *auris*, the MFCs of *C*. *krusei* were high with TM4 and TM5 (>64 and 64 μg/mL (51.2 and 76.8 μM, respectively)), but TM9 had an MFC of 16, with *C*. *krusei*. *C*. *glabrata* was most sensitive to TM4, with an MIC of 8 μg/mL (6.4 μM). *C*. *parapsilosis* was most sensitive to TM4 and TM5, with an MIC of 8 μg/mL (6.4 and 9.6 μM). TM4 gave an MFC of 16 μg/mL (12.8 μM) and TM5 gave an MFC of 32 μg/mL (38.4 μM) against *C*. *parapsilosis*. *C*. *albicans* was sensitive to all peptoids with an MIC of 8 μg/mL (6.4, 9.6, and 6.2 μM). The MFCs for *C*. *albicans* ranged from 16 (TM4) to 64 μg/mL (TM9) (12.8 to 49.6 μM).

**Table 2:** Minimum Inhibitory Concentration (MIC) and Minimum Fungicidal Concentrations (MFC) for Peptoids TM4, TM5, TM9, and LL-37 against Different *Candida* Species^a^

Species	*C. albicans*		*C. glabrata*		*C. dublinensis*		*C. krusei*		*C. parapsilosis*	
Peptoid	MIC	MFC	MIC	MFC	MIC	MFC	MIC	MFC	MIC	MFC
*TM*4	8	16	8	8	16	32	0.0625	>64	8	16
*TM*5	8	32	16	32	64	>64	0.025	64	8	32
*TM*9	8	64	64	64	64	>64	2	16	64	64
*LL-*37	>64	>64	ND	ND	>64	>64	>64	>64	ND	ND

a Data are shown in μg/mL. ND, not determined.

TM5, which is the peptoid used in the *in vivo* experiment, exhibited activity against *C*. *albicans, C*. *glabrata, C*. *dubliniensis*, *C*. *krusei*, and *C*. *parapsilosis,* ranging from 0.025 to 16 μg/mL (0.024–19.2 μM), except the MIC against *C*. *dubliniensis* was high at 64 μg/mL. However, the MFCs of TM5 ranged from 32 to 64 μg/mL (38.4–76.8 μM), except there was no MFC against *C*. *dubliniensis* (>64 μg/mL). Due to the variable activity of TM9, we continued the study with TM4 and TM5.

## Effect of Cations on MIC of Peptoids

We have previously demonstrated that the initial interaction of cationic AMPs and mimetics with the fungal membrane is electrostatic. When Na^+^, Ca^2+^ and La^+++^ ions were added to the MIC assays, there was a valence-dependent inhibition of anti-*Candida* activity.^35^ Furthermore, we recently observed an inhibition of antiviral activity of an antimicrobial peptoid by cationic delivery vehicles.^36^ To determine if the cationic peptoids exhibit a similar interaction with the *C*. *auris* membrane, we added CaCl_2_ or MgCl_2_ to the MIC assay for TM5 at increasing concentrations from 0 to 20 mM. While both cations led to a reduction in activity as evidenced by an increase in MIC, addition of CaCl_2_ led to a complete inhibition (MIC > 64 μg/mL) at 5 mM, but MgCl_2_ only increased the MIC by 2-fold even up to 20 mM (Table 3).

**Table 3:** Effect of Divalent Cations on TM4 and TM5 Peptoid Activity Against *C*. *auris*

Fold increase in MIC due to divalent cations:		
TM4	Ca^2+^	Mg^2+^
2.5 mM	0	0
5 mM	>4	2
10 mM	>4	2
20 mM	>4	2
TM5	Ca^2+^	Mg^2+^
2.5 mM	0	0
5 mM	>4	2
10 mM	>4	2
20 mM	>4	2

## Temporal Fungicidal Activity

Figure 1 shows the kinetics of TM5 compared with caspofungin against *C*. *auris* strain 381. Caspofungin and TM5 were tested at two concentrations: 5 and 10 times the MIC. The cytotoxicity kinetics of TM5 were 25 times faster than caspofungin on *C*. *auris* strain 381 at both concentrations. TM5 completely killed all cells within 1 h at both concentrations, whereas only 40% were killed at 1 h with caspofungin and never reached complete killing at 24 h. Similar experiments were done with *C*. *auris* strains 382, 383, and 384 with both TM4 and TM5 at 5 times and 10 times the MIC. A 50% reduction of O.D. was observed in 15 min (data not shown).

**Figure 1:**
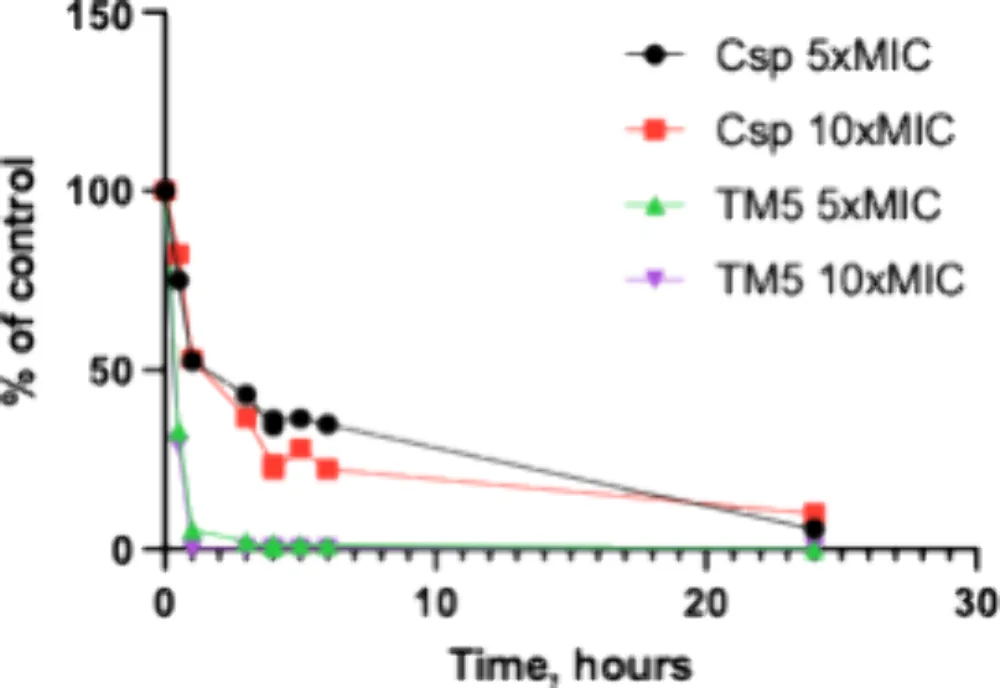
Kinetics of antifungal activity of caspofungin and TM5. *C*. *auris* strain 381 was grown in the presence of 5X or 10X MIC of caspofungin (Csp) or TM5 and plated on YPD to quantify viable cfu.

## Antifungal Resistance

A large problem in treatment of fungal diseases is the development of resistance of *C*. *auris* to antifungal drugs, especially the azoles. Therefore, a resistance study was performed. Figure 2 shows that *C*. *auris* strain 381 does not develop resistance to TM4 and TM5. After daily passages for 20 days, the MIC of TM5 remained unchanged using *C*. *auris* strain 381 (original MIC at day 0 was 4 μg/mL (4.8 μM)). In contrast, *C*. *auris* strain 381 passaged 20 times each day in fluconazole increased to 8 times the original MIC, 16 μg/mL (19.2 μM) in this experiment, at day 0. A lack of resistance development was also observed treating *C*. *auris* strain 381 with TM4.

**Figure 2:**
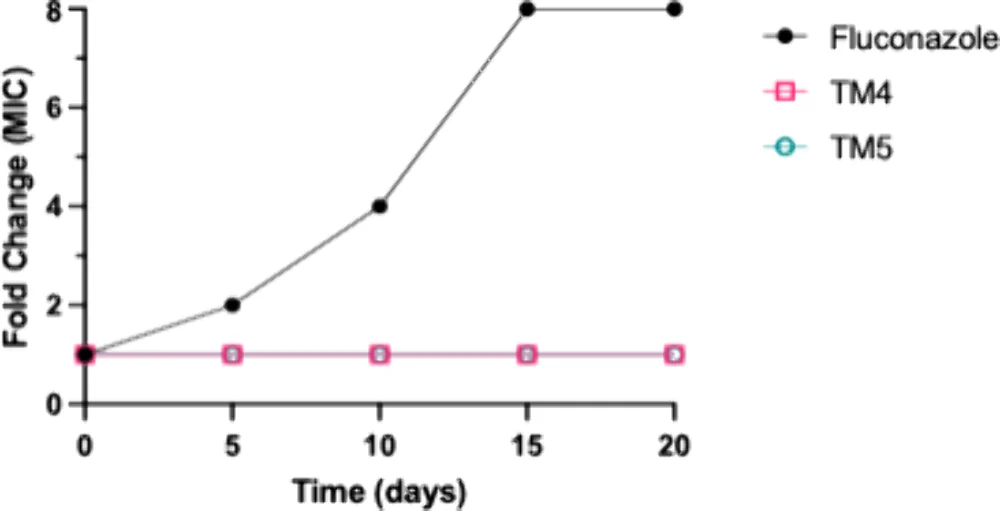
*C*. *auris* does not develop resistance to TM4 and TM5. *C*. *auris* strain 381 was grown and passaged daily in the presence of 0.5X MIC TM4, TM5 or fluconazole. Samples were taken every 5 days and assessed for change in the MIC.

## Synergy with Standard Antifungal Drugs

The ability of peptoids to be more effective when combined with existing antifungal drugs was tested. We carried out standard checkerboard synergy assays to quantify the synergy between TM4 and TM5 with representative antifungal drugs. Fractional inhibitory concentration index (FICI) was calculated to determine synergy, where an FICI of <0.5 is defined as synergistic, and an FICI of 0.5–1.0 is defined as additive. Table 4 shows that TM4 combined with fluconazole leads to a FICI of 0.375, indicating strong synergy in killing *C*. *auris* strain 381. Even stronger synergy was observed with amphotericin B (FICI = 0.25). TM4 had an additive effect with caspofungin with a FICI of 0.75. TM5 with fluconazole also indicates borderline synergy with a FICI of 0.5. There was strong synergy with both caspofungin, giving a FICI of 0.25, and amphotericin B (0.1875).

**Table 4:** Fractional Inhibitory Concentration Index (FICI) of TM4 and TM5 with the Standard Antifungal Drugs Fluconazole, Caspofungin, and Amphotericin B on *Candida auris* Strain 381

	Fluconazole	Caspofungin	Amphotericin B
TM4	0.375	0.75	0.25
TM5	0.5	0.28	0.1875

## Fluorescence Microscopy Imaging of Peptoid Killing of *C*. *auris*

To observe the effect of TM5 on cellular structure, we treated C. auris 381 with TM5, caspofungin or fluconazole followed by staining with calcofluor white (Figure 3). Calcofluor white stains chitin and cellulose on the cell wall of *C*. *auris*, showing a ring-like image of the yeast cell in untreated control samples. The yeast cells, which were not aggregated or filamentous, were exposed for 1 or 2 h to 40 μg/mL (48 μM) TM5. The results show fluorescence throughout the TM5-treated cells after 2 h, suggesting that a disruption in the membrane allows the migration of the chitin from the cell wall into the cell. Fluconazole and caspofungin at 10X MIC treatment show little, if any, effect at 2 h and the images resemble untreated yeast cells.

**Figure 3:**
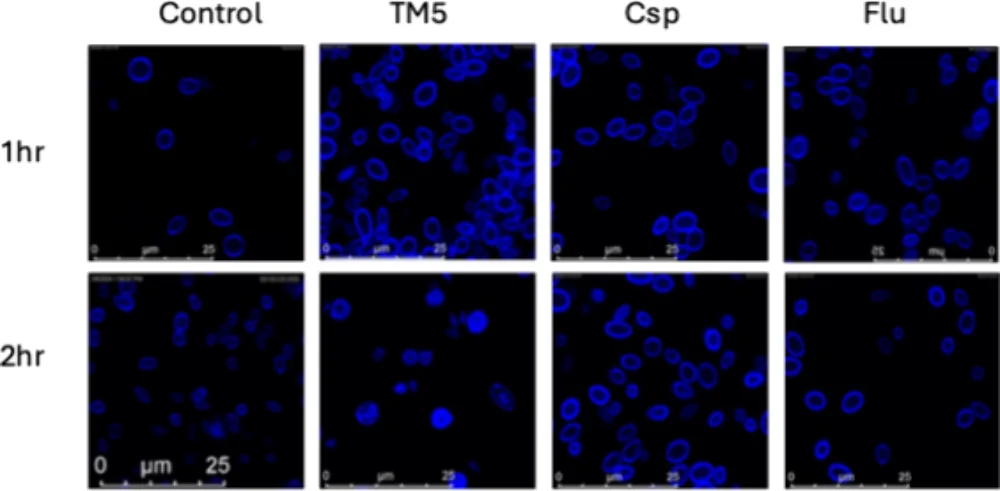
Membrane disruption of *C*. *auris* by TM5 as demonstrated by fluorescence microscopy. *C*. *auris* untreated or treated for 1 or 2 h with 40 μg/mL (48 μM, 10X MIC) TM5; or with 10X MIC of caspofungin (D) or 10X MIC of fluconazole. Samples were stained with calcofluor white and visualized by fluorescence microscopy.

## TEM Imaging of Peptoid Treatment of *C*. *auris*

Visualization of peptoid killing of *C*. *auris* by TEM is depicted in Figure 4. Untreated *C*. *auris* strain 381 shows a defined cell membrane, a defined cell wall, and round defined nucleus and organelles. However, outer membrane disruption of the yeast cell is clearly observed in as little as 2 h after TM5 treatment at 10X MIC. In addition, after 2 h, the organelles and nucleus started to lose their definition. By 24 h, the membrane was completely dissolved and the organelles inside the cell show aggregation and appear scrambled. The nuclear membrane also appeared to be completely disrupted by 24 h.

**Figure 4:**
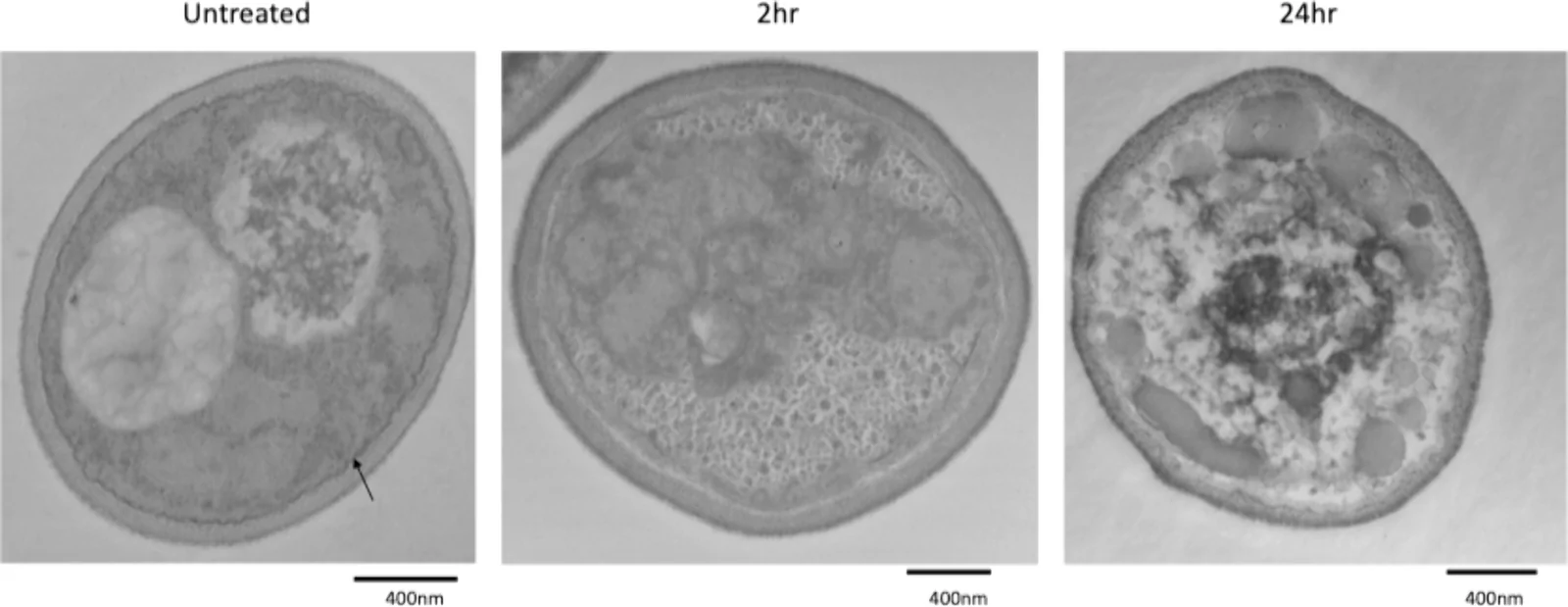
Membrane disruption of *C*. *auris* by TM5 visualized by transmission electron microscopy (TEM). *C*. *auris* strain 381, either untreated, or treated with 10X MIC TM5 for 2 or 24 h followed by fixation was visualized by TEM. Arrow shows the intact fungal membrane in the untreated sample.

### *In Vivo* Activity of TM5 against *C*. *auris*

Figure 5 depicts the dose–response of cfu/g kidney versus dose of TM5 in an immunocompetent C57Bl/6 mouse model of *C*. *auris* 381 candidemia, a systemic blood infection. The mean amount in untreated, control infected mice (PBS) was 4 × 10^5^ cfu/g kidney. A previous study indicated that the maximal tolerated dose (MTD) for TM5 was 5 mg/kg (K. Kirshenbaum, personal communication). A subcutaneous injection of TM5 dissolved in PBS 3 h after intravenous infection at doses 1–5 mg/kg was effective in eliminating the kidney burden of yeast. After 24 h the kidney was excised and there were no colonies on the YPD agar at a 1:10 dilution of the homogenate at 2.5 mg/kg and 5 mg/kg, except one outlier at 5 mg/kg. The mice showed no side effects from the drug, based on clinical scoring, nor was any change in weight observed. At 1 mg/kg, more than half of the cfu were recorded, showing that as little as 1 mg/kg was effective in eliminating a significant portion of the *C*. *auris* strain 381 yeast cells.

**Figure 5:**
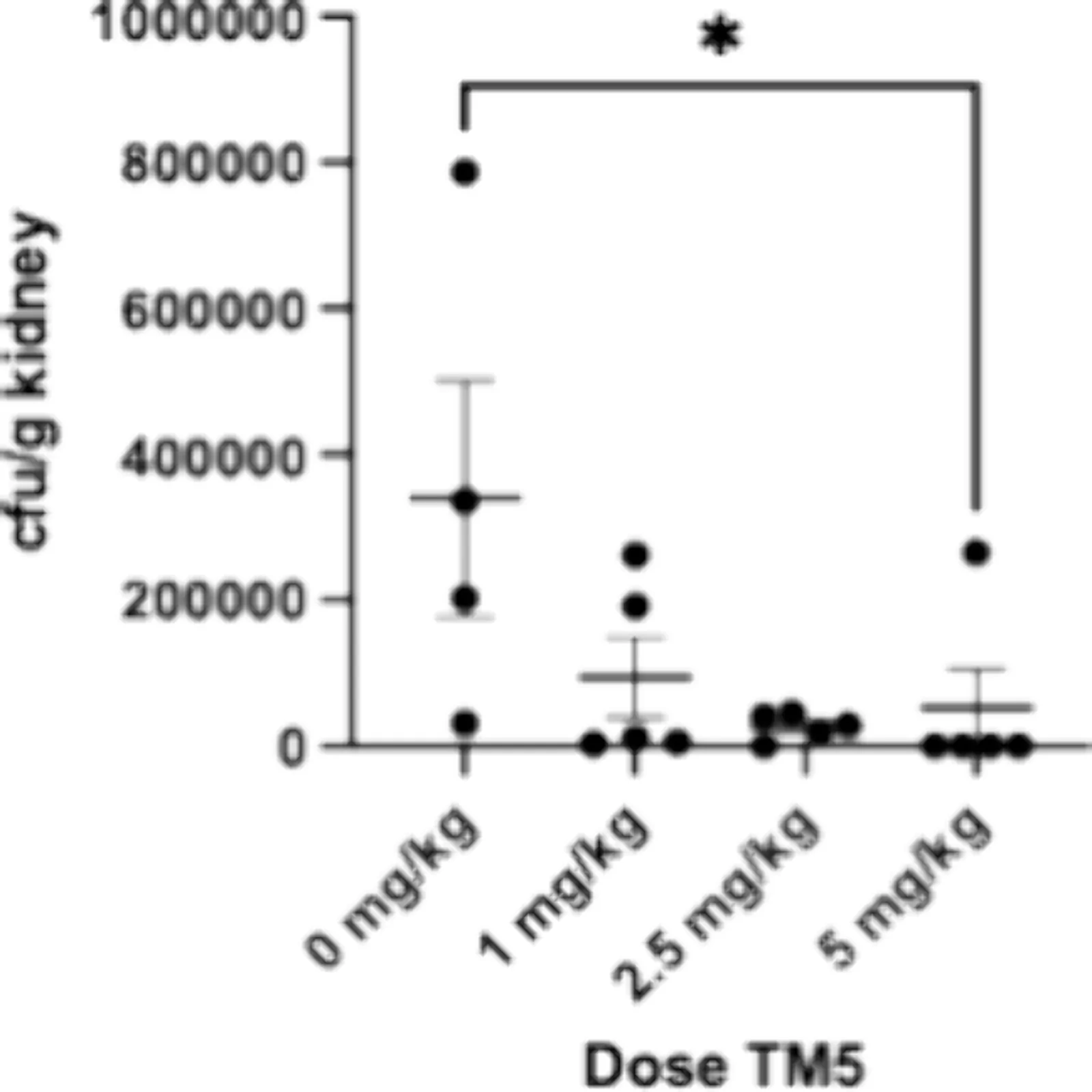
*In vivo* activity of TM5 in a systemic candidiasis model of *C*. *auris* infection. *C*. *auris* strain 381 (1 × 10^9^ cfu/mL) was injected into mice (*n* = 20) by tail vein injection (0.1 mL). At 3 h post infection, mice were injected subcutaneously with TM5 in PBS (*n* = 5 per dose). At 24 h post infection, mice were euthanized, kidneys were excised and homogenized in PBS. Homogenates were diluted and viable cfu were enumerated by plating dilutions on YPD in duplicate. *, *p* ≤ 0.05 by *t*-test.

In a separate experiment, mice were infected with *C*. *auris* 381 as above and treated with TM5 dissolved in 20% kleptose (β-cyclodextrin), which is commonly used as a drug-delivery vehicle to enhance bioavailability. Surprisingly we observed no reduction in cfu with this solution (data not shown).

## Discussion

This is the first report of dose-dependent systemic peptoid activity against a microorganism *in vivo* in an animal model. In addition, are no other reports of *in vivo* activity, even on the mucosal surfaces of animal models of other *Candida* species, including *C*. *auris*. In this study, the infection was delivered intravenously and *C*. *auris* strain 381, which was in nonaggregated form, as indicated by Phloxin B staining,^37^ traveled through the blood to the kidney, where the yeast colonies from the homogenates were detected on YPD agar. While TM5 has a MFC of 64 μg/mL (76.8 μM), its MIC is 4 μg/mL (4.8 μM) against *C*. *auris* strain 381 and it is reported to lack toxicity in the lung,^19^ suggesting that TM5 was one of the best antifungal agents to test against *C*. *auris* strain 381. Thus, this was the rationale for testing this peptoid *in vivo*. Subcutaneous injection was chosen as the delivery method to further limit potential toxicity in this candidemia model. Delivery at 3 h after infection was used to target the yeast as it was distributing throughout the body via the bloodstream.

No detection of *C*. *auris* was observed in the 1:10 diluted kidney homogenate (lowest dilution) when treated with TM5, indicating that the subcutaneous delivery of TM5 was able to completely kill the circulating yeast cells and led to a 5-log reduction in colony forming units compared with PBS treatment controls. No overt toxicity was observed in the mice during the 24-h experiment in mice treated only with TM5 in PBS. The mice remained alert, and overall observations of sickness were not observed, such as hair ruffling, lethargy, and isolation behavior. In addition, in a separate, uninfected group that received only 5 mg/kg TM5, the mice did not lose weight over 1 week compared to the group that received only PBS (data not shown). During the 24-h infection, the kidney weights also did not change significantly when the drugged groups were compared to the PBS group. More comprehensive toxicity studies are warranted to establish a safety profile for the subcutaneous delivery of peptoids in this *C*. *auris* infection model. However, *in vitro* toxicity studies were done against human primary EpiAirway cells with TM5 with no dose-dependent toxicity on mitochondrial activity as indicated by the MTT assay.^26^ No hemolysis studies were done using TM5.^33^ Spheroid models of A549 human lung epithelial cells showed decreased cytotoxicity with TM5 treatment, with only minimal cell death observed at a TM5 concentration of 240 μg/mL (288 μM).^19^

The structure of the TM5 peptoid has been published previously^38^ and as MXB5.^26,28^ The dissolution TM5 in PBS was favored to encourage the formation of ellipsoid micelles of peptoid as needed to penetrate the membrane of the yeast cell.^33,38^ Based on the structure of kleptose, we hypothesize that the formation of self-assembled TM5 micelles that is necessary to penetrate the membrane of *C*. *auris* may have been impeded when TM5 was dissolved in 20% kleptose, leading to little fungicidal killing of *C*. *auris in vivo* when kleptose was used in dissolution. This indicates that the vehicle choice for formulation of this antifungal treatment is critical, and that the vehicle cannot contain molecules or surfactants that may interfere with micelle formation. It is advantageous that TM5 is soluble in PBS for easy subcutaneous delivery. In addition, the killing of *C*. *auris* in this model indicates that TM5 is not inactivated by serum in the bloodstream, a feature that is an improvement compared with AMPs, which are inactivated. It has been established that peptoids are not inactivated by proteases due to their structure.^15^

Other *in vivo* studies show efficacy of various peptoids with different animal models of infectious diseases. These studies also highlighted the lack of toxicity both *in vitro* and *in vivo*. In a mouse animal model of intraperitoneal *Staphylococcus aureus* infection, 4 mg/mL of peptoid 1, administered locally, significantly decreased the number of bacteria in the peritoneal lavage fluid by 2 logs compared with saline.^39^ In a murine incision wound model, 100 μM peptoid 1 in PBS was able to decrease bioluminescent *S*. *aureus* to undetectable imaging flux levels compared with PBS 8 days after infection.^20^ *In vivo* activity of peptoids has been demonstrated against ESKAPE bacteria such as *Pseudomonas aeruginosa* and *S*. *aureus*-induced skin abscesses in mice.^38^ In addition, peptoid TM18 and TM1 were tested on the mouse skin abscess model against dual species infections with *S*. *aureus* and *P*. *aeruginosa* and were found to significantly reduce abscesses, bacterial load and increase survival.^40^ In contrast to our model, these models were not systemic infections but demonstrated the local efficacy and broad-spectrum activity of the antimicrobial peptoids. In the Nielsen et al. model, for example, peptoids with different structures had a varying effect on the magnitude of effectiveness, with peptoids TM2, TM5 and TM8 having no effect on *P*. *aeruginosa* abscesses or bacterial load and peptoid TM4 reducing abscess size but not bacterial load. However, peptoid TM1 was able to reduce the *P*. *aeruginosa* bacterial load in the abscess 5-fold and the abscess size 2-fold. TM5, the peptoid used in our study, reduced *S*. *aureus* abscess size by 80% and bacterial burden by 1,000-fold. Peptoids TM1 and TM2 were most effective on *S*. *aureus*, with bacterial load reductions of 10,000-fold and 100,000-fold, respectively, and reductions in abscess size by 90%. In a mouse model of a lethal *P*. *aeruginosa* Xen41 intratracheal infection of the lung, 40 μg/mL (48 μM; 5x MIC) and 80 μg/mL (96 μM; 10x MIC) TM5 significantly reduced the bacterial burden in the lungs, increased survival to 100%, and increased neutrophil and inflammation in histological lung sections compared with PBS treatment.^19^ We did not compare other peptoids to TM5 in our *in vivo* systemic model of *C*. *auris* infection due to the effectiveness of TM5. However, it would be interesting to examine structure and *in vivo* fungicidal activity of various peptoid structures to find the optimal SR and therapeutic index (TI) with *C*. *auris*.

Even though the TM5 structure design was inspired by LL-37 self-assembly and pan-antimicrobial activity, the MICs and MFCs of this peptoid against *C*. *auris* were very different than those of LL-37. Whereas the MIC of LL-37 against *C*. *auris* strain 381 was >64 μg/mL (14.4 μm), the MIC of TM5 was 4 μg/mL (4.8 μM). The MFC was greater than 16-fold higher, indicating that greater amounts of TM5 were required for complete killing of the yeast. Therefore, LL-37 is not effective on *C*. *auris in vitro* so *in vivo* systemic studies with LL-37 were not performed. Although LL-37 is reported to be candidacidal, severely affecting membrane morphology and associating with the cell wall mannan,^41,42^ in our repeated experiments, LL-37 MIC and MFC on *C*. *albicans* was >64 μg/mL (14.4 μM), making it ineffective against *C*. *albicans*. This result is strengthened by a comparison between LL-37 and TM5 against *P*. *aeruginosa* and *S*. *aureus*, both in planktonic bacteria and in biofilms.^43^ In this study, LL-37 failed to inhibit the bacterial growth at concentrations >64 μg/mL (14.4 μM), comparable to the reported MIC of >100 μg/mL (22.5 μM),^25,44^ whereas TM5 inhibited *P*. *aeruginosa* (PAO1) and *S*. *aureus* (SA25923) with MICs of 8 and 16 μg/mL (9.6 μM and 19.2 μM), respectively. Biofilms of these bacteria were eradicated by TM5 (70 μg/mL (84 μM) for both bacteria), but LL-37 did not eradicate both types of biofilms (>200 μg/mL; 45 μM).

When comparing TM5 to our published peptide foldamer, peptoids were much more active on a per weight basis. These peptide mimetics, based on magainin, were very effective against *C*. *albicans* in both oral and systemic models in higher doses below the MTD.^35,45–47^ *In vivo*, the peptide mimetic from these published studies was not comparable to TM5 against *C*. *auris*, as 5 mg/kg only achieved a 0.5 log drop in cfu/g kidney compared to the peptide mimetic vehicle (20% kleptose in PBS to dissolve this drug) (data not shown), whereas 5 mg/kg TM5 in 0.1 mL PBS dropped the cfu/g kidney to undetectable levels in as little as 24 h (peptoid TM5 is soluble in aqueous solutions).

Considering the mechanistic studies of these peptoids against different species of *Candida* and different clades of *C*. *auris*, including fluconazole-resistant strains, the peptoids are quite effective in disrupting the membrane of the yeast cell without impacting the cell wall. The results demonstrated this membrane disruption with the representative TEM and fluorescence micrographs of *C*. *auris* strain 381 in Figures 3 and 4. Others have also shown that the action of the peptoids on *Candida* species is on the membrane and not the cell wall, as indicated by the lack of an MIC increase in the sorbitol protection assay compared with caspofungin.^33^ The action is very quick compared to caspofungin, as 10x MIC (40 μg/mL (48 μM)) of TM5 began to kill the yeast cells within 15 min of incubation and killed all cells after 1 h. In contrast, 10x MIC of caspofungin killed 50% of the yeast after 1 h and never killed all the cells after 24 h. Others have found that peptoids kill *Candida* as quickly.^32,33^ The swiftness of the peptoids in killing microorganisms is an advantage over conventional antifungal drugs and can avoid potential toxicity and development of resistance. Following disruption of the *C*. *auris* membrane, the TEM micrographs show that the organelles conglomerate, presumably via disruption of organelle and nuclear membranes occurring within 2 h of peptoid exposure *in vitro*.^30^ The results showing the inhibition of activity by calcium and to a lesser extent by magnesium are similar to those observed with the antifungal peptide cryptomycin.^48^ This suggests that there may be an additional mechanism involving calcium homeostasis in addition to the activity on the membranes. Another observation of an alternative mechanism of action is indicated by the *in vitro* MIC results with *C*. *krusei*, which are below 1 μg/mL (1.1 μM) with TM4 and TM5 peptoids. This may indicate that the mechanism of action of the peptoids may have switched from a nonspecific mode of action (membrane active, nucleic acid interactions) to a specific mode of action (engaging with a particular enzyme or other essential protein target) in that particular *Candida* species. This should be investigated further.

Our results suggest that these antimicrobial peptoids could be a useful antifungal treatment, either alone or in combination with existing antifungal drugs, as indicated by the synergistic antifungal drug results, particularly as an alternative therapy for antifungal-resistant strains of *C*. *auris*. Our results show that *C*. *auris* strain 381 resistance to TM5 is difficult to induce compared with fluconazole, even after 20 days. The peptoids can be synthesized in bulk and for little cost, making drug development feasible. Further clinical development of the peptoids studied here could lead to a new class of antifungal drugs that are slow to develop resistance and have low toxicity, possibly reducing the mortality against systemic candidiasis.

## Materials and Methods

### Reagents

Peptoid compounds were synthesized, purified and characterized as described^16,26,28^ and dissolved in phosphate-buffered saline (PBS) (Mediatech, Inc., Manassas, VA, USA) at a stock concentration of 2 mg/mL and stored at −20 °C. Caspofungin, fluconazole, RPMI 1640 containing l-glutamine without bicarbonate, buffered to pH 7.0 with 0.165 morpholinepropanesulfonic acid (MOPS) buffer were purchased from Sigma-Aldrich, St. Louis, MO, USA. The EM fixative, 2.5% glutaraldehyde in 0.1 M cacodylate pH 7.4, was also purchased from Sigma. Calcofluor-white bis­(1,3-dibutylbarbituric acid) trimethine oxonol was purchased from Life Technologies, Eugene, OR, USA. LL-37 was obtained from Anaspec, Fremont, CA, and tested for activity against HSV-1 in an antiviral assay.^26^

### Yeast Strains

Clinical isolates of *Candida auris* were obtained from the FDA-CDC Antimicrobial Resistance Isolate Bank (Panel Details accessed May 7, 2026). CDC strains 381–390 are from four major clades; CDC strains 382, 387, 388, 389, and 390 are from Clade I (South Asia), CDC strain 381 is from Clade II (East Asia), CDC strains 383 and 384 are from Clade III (South Africa), and CDC strains 385 and 386 are from Clade IV (South America)). CDC strains 381, 382, and 387 are sensitive to antifungal drugs. CDC strains 383, 384, 385, 386, 388, 389, and 390 are fluconazole-resistant *C*. *auris* strains (MIC ≥ 64). All strains were echinocandin-sensitive. The *C*. *auris* isolates were cultured on YPD (1% yeast extract, 2% peptone, and 2% dextrose) agar at 37 °C and used for peptide mimetic compounds investigation. Other species of *Candida (C*. *albicans*, *C*. *dublineinsis*, *C*. *glabrata*, *C*. *krusei*, and *C*. *parapsilosis*) were obtained from ATCC, and were cultured using the same YPD agar but incubated at 30 °C.

### MIC/MFC Assays

Minimum inhibitory concentration (MIC) and minimum fungicidal concentration (MFC) were quantified according to CLSI methods as previously described.^46^ Ten *Candida auris* strains and four other species of *Candida* were tested against three different peptoid compounds. Peptoid compounds at a concentration 256 μg/mL were diluted in 50 μL RPMI/MOPS cell culture medium to a final concentration of 64 μg/mL in 96-well plates (tissue culture treated; Costar, Corning Incorporated, Corning, NY, USA). Optical densities of fungus suspensions in phosphate-buffered saline (PBS) were adjusted to an OD_600_ of 1.0. This suspension was further diluted to 1:100 in RPMI/MOPS buffer. After serially diluting peptoid compounds, 50 μL of *Candida* was added to every well. Plates of *Candida auris* were incubated at 37 °C for 24 h while other *Candida* species were incubated at 30 °C for 48 h. After the incubation period, 10 μL of solution considered to be the MIC and three wells of ascending concentration were plated on YPD agar to incubate for another 24 h. Colony presence for each compound-fungus strain combination was noted after incubation, where the MFC is defined as having no existence of colony observed. All combinations of peptide mimetic compound and experimental fungus strain were performed in duplicate for each MIC/MFC assay.

### Fungicidal Kinetics on *C*. *auris*

Time-kill kinetics of TM5 and caspofungin were measured using *Candida auris* (strains 381 and 382). *Candida* was suspended in PBS and adjusted to 1 mL of concentration of 2 × 10^4^ cfu/mL. The suspensions were treated with 5X or 10X MIC of compound and incubated at 37 °C. 10 μL aliquots were removed at 15′, 30′, 1 h, 3 h, 4 h, 5, 6, and 24 h, diluted in 1 mL PBS and plated on YPD to quantify viable colonies.

### Resistance Studies

*C*. *auris* strain 381 was passaged daily in 0.5X MIC of either fluconazole or TM5 in 5 mL YPD for 20 days. Samples were taken every 5 days and plated on YPD agar to obtain single colonies. The MIC of these samples was quantified in duplicate as described above. Fold change from the initial MIC was determined over the 20-day period.

### Synergy Assays

*Candida auris* (strain 381) was used to measure synergies of peptoid compounds TM4 and TM5 with the established antifungal drugs caspofungin and fluconazole. In separate 96-well plates, all four compounds at concentration 64 μg/mL were diluted in 50 μL RPMI/MOPS cell culture medium to a final concentration of 16 μg/mL. Optical densities of *C*. *auris* strain 381 suspensions in PBS were adjusted to an OD_600_ of 1.0. This suspension was further diluted to 1:100 in RPMI/MOPS buffer. After serial 2-fold dilutions to 0.25 μg/mL, the peptoid plates were crossed with plates of either caspofungin, fluconazole or amphotericin B in a checkerboard assay. Fifty μL of the *Candida* suspension containing the peptoid was then added to every well, and plates were incubated at 37 °C for 24 h. The fractional inhibitory concentration index (FICI) was then determined using the following equation FICI = (MIC_AB_/MIC_A_) + (MIC_BA_/MIC_B_). A FICI of <0.5 is defined as synergistic. A FICI of 0.5–1.0 is defined as additive.

### Electron Microscopy

Transmission electron microscopy (TEM) images of *Candida auris* (strain 381) were captured before and after treatment with TM5. Fungal suspensions in PBS were adjusted to an OD_600_ of 6.0 and divided equally into three tubes, diluted to 1 mL with PBS each. TM5 at 10X MIC concentration was added to each of two tubes and incubated at 2 and 24 h, respectively. After 2 h, treated and control samples were centrifuged at 1,000 rpm for 10 min, washed with PBS, and fixed in 1 mL 2.5% glutaraldehyde in 0.1 M cacodylate pH 7.4 to be stored at 4 °C until TEM was performed 24 h later. These steps were repeated for the 24-h suspension.

### Fluorescence Microscopy

*Candida auris* (strains 381 and 382) in PBS suspension were adjusted to an OD_600_ of 6.0 and divided equally into five tubes of 450 μL PBS. TM5 at 10X MIC concentration was added to each of four tubes and incubated at 15 min, 45 min, 2 h, and 24 h, respectively, in a 37 °C water bath. Cells were then fixed with 50 μL 36% formaldehyde (Supelco) for 30 min and washed twice with PBS (6,000 rpm for 5 min). *C*. *auris* was then resuspended in 50 μL calcofluor white (Sigma) for 5 min, washed 5 times with PBS, and treated with 50 μL 10% KOH. Specimens were plated on slides and observed on a Leica 6000 Confocal Microscope. This process was repeated for treatment of 10x MIC of caspofungin and 10x MIC of fluconazole for comparison as well as an untreated control.

### *In Vivo* Efficacy Studies

Female mice (C57BL/6), aged 6–8 weeks, were procured from Charles River Laboratories and allowed to acclimate 1 week under specific pathogen-free conditions in a barrier facility and fed a standard laboratory rodent diet. The study adhered to protocols approved by the Institutional Animal Care and Use Committee (IACUC) at the University of Louisville (Protocol number 23277). Groups of five C57Bl/6 female mice, 6–8 weeks old, were intravenously injected with 1 × 10^5^, 1 × 10^6^, 1 × 10^7^, 1 × 10^8^ and 1 × 10^9^ per 0.1 mL PBS of *C*. *auris*. Inoculum was confirmed following dilution, duplicate plating of each dilution and counting colonies grown on YPD agar. Without immunosuppression, it was determined that further infections would use 1 × 10^9^/0.1 mL/mouse to yield an appropriate kidney burden for antifungal drug testing. After 2 h following infection, groups of 5 mice were injected subcutaneously with dilutions of TM5 in PBS. As an alternative, mice were injected subcutaneously with dilutions of the peptoid solution in 20% kleptose in PBS to determine the effect of the vehicle. Following 24 h, mice were sacrificed using CO_2_ asphyxiation. Due to the stability and high burden of yeast cells in the kidney, which is most representative of systemic disease,^49^ only kidneys were excised using sterile technique and homogenized in 4.0 mL sterile PBS. Dilutions of homogenates were plated (0.1 mL) on YPD agar in duplicate and grown at 37 °C for 48 h prior to counting (30–300 colonies per plate).

## Abbreviations

AMP: antimicrobial peptide
FICI: fractional inhibitory concentration index
HNP-1: human neutrophil peptide
hBD-3: human beta-defensin-3
MIC: minimum inhibitory concentration
MFC: minimum fungicidal concentration
MRSA: methicillin-resistant *Staphylococcus aureus*
MW: molecular weight
PBS: phosphate buffered saline
SR: selectivity ratio
MTD: maximum tolerated dose
TI: therapeutic index
XDR: extreme drug resistance
YPD: yeast peptone dextrose
